# The Nurse Education and Transition (NEAT) model: educating the hospitalized patient with diabetes

**DOI:** 10.1186/s40842-016-0020-1

**Published:** 2016-01-14

**Authors:** Jodi Stotts Krall, Amy Calabrese Donihi, Mary Hatam, Janice Koshinsky, Linda Siminerio

**Affiliations:** 1 University of Pittsburgh Diabetes Institute, 4401 Penn Avenue, LMB Room 2112, Pittsburgh, PA 15224 USA; 2 School of Pharmacy, University of Pittsburgh, Pittsburgh, PA USA; 3 University of Pittsburgh Medical Center, Pittsburgh, PA USA; 4 Department of Medicine, University of Pittsburgh, Pittsburgh, PA USA

**Keywords:** Education, Hospital, Inpatient, Nurse

## Abstract

**Background:**

The number of patients with a diabetes mellitus (DM)-related diagnosis is increasing, yet the number of hospital-based diabetes educators is being reduced. Interest in determining effective ways for staff nurses to deliver diabetes education (DE) is mounting. The purpose of this multi-phase feasibility study was to develop and evaluate the Nurse Education and Transition (NEAT) inpatient DM education model.

**Methods:**

Exploratory focus groups were conducted with staff nurses from inpatient units at academic tertiary and community hospitals to gain insight into barriers, content, delivery and support mechanisms related to providing DE to hospitalized patients. Findings informed the development of the NEAT model, which included a delivery protocol and toolkit with brief educational videos on key diabetes topics uploaded onto iPads, patient assessments and “teach back” tools, a discharge survival skills summary sheet, and guidelines for electronic medical record documentation and scheduling outpatient DE visits. Trained staff nurses used NEAT to deliver DE to hospitalized patients with DM and then participated in follow-up focus groups to assess their experiences, with particular attention to the usefulness of NEAT in meeting the needs of nurses related to the delivery of diabetes survival skill education. Information generated was analyzed to identify emerging key themes.

**Results:**

Exploratory focus groups revealed that staff nurses view teaching patients with DM as part of their job, but report barriers. Nurses agreed that inpatient DE should be designed to assure safety after discharge and advised that it be patient-centered, targeted, assessment-based and user friendly. Nurses who participated in the delivery of NEAT found that the process and tools met the majority of the basic DE needs of their patients while relieving their workload. In particular, they reported that video and iPad technology provided a convenient and standardized method for facilitating teaching at the bedside, but requested that an interactive feedback mechanism be added to encourage patient self-knowledge assessment.

**Conclusions:**

This study presents challenges staff nurses face in providing DE to hospitalized patients and identifies opportunities and strategies for improving content and delivery to ensure safe transition of patients with DM from hospital to outpatient setting.

## Background

People with diabetes mellitus (DM) are more likely to be hospitalized and have longer length of stay (LOS) than those without DM [1]. A recent survey estimated that 22 % of all hospital inpatient days were incurred by people with DM [2] and that inpatient care accounts for 43 % of the total medical costs of diabetes [3]. Because the proportion of hospitalized patients with diabetes has risen steadily over recent decades in tandem with the increasing incidence of diabetes in the general population [4], these rates may continue to climb [5]. It is no surprise that interest is growing in determining ways to improve hospital management with evidence-based protocols [6–8] and a Transition of Care Coalition [9], established to develop approaches to assure a safe transition following discharge. The Joint Commission on Hospital Accreditation and the American Diabetes Association (ADA) recommend that inpatient programs specifically include staff training and patient self-management education [9].

DM is a complex chronic disease that requires the person with DM to make a multitude of daily self-management decisions and perform numerous care activities. After a hospital stay, patients often face added challenges regarding diabetes management. Some patients may receive a new diagnosis of DM while those diagnosed are likely to have their treatment plans adapted. For example, those formerly taking oral anti-hyperglycemic medications may start insulin injections, or have their monitoring, activity or nutrition plans changed, all of which require extensive education so that a patient can self-manage their condition upon discharge [10].

Diabetes self-management education (DSME) provides the foundation for self-care and helps people living with DM navigate daily decisions and activities. DSME has been shown to improve health outcomes and reduce hospital readmissions and costs [11–14]. It is recommended that all patients with DM receive DSME [10]. An audience of hospitalized patients offers an opportunity to reach people with DSME and for many years diabetes educators were employed to provide this inpatient service.

However, it has been shown that attempts in providing comprehensive DSME during a hospital stay are often ineffective for a number of reasons [15, 16]. A hospital stay does not always afford a “teachable” moment,” given hospitalized patients are acutely ill and have competing demands like scheduled procedures [15]. The increasing number of people with DM [4] and limited hospital length of stay (LOS) [17], presents additional challenges for providing comprehensive DSME to a large number of patients during a brief hospital admission. Nevertheless, educating hospitalized patients is considered to be important, and many institutions are re-examining the delivery during a hospital stay and the role of dedicated educators to provide this service. More recently, health systems are adopting models where diabetes education in the hospital solely focuses on survival skills, including hypoglycemia, medication education, nutrition, and blood glucose monitoring [15, 18, 19], and detailed DSME is typically deferred to the outpatient arena.

Hospital bedside nurses are expected to provide the “survival skills” education, but are often unprepared and overwhelmed with many other responsibilities. As a result, education tends to be inadequate and fragmented. Patients often leave the hospital without the self-care skills and follow up referral to DSME that can result in subsequent problems, like readmission [18]. Thus, interest in determining effective ways for staff nurses to provide basic DSME education and address transition on discharge is mounting. Despite reports on effective inpatient education programs [17, 20–22], no standardized, evidence-based programs have been developed for training bedside nurses in DM education and transition.

The objectives of this multi-phase, feasibility study were to (1) explore staff nurse perceptions of their role and experiences in providing education to hospitalized patients with DM, and, based on these findings, (2) develop and assess the feasibility of the Nurse Education and Transition (NEAT) inpatient DM education model.

## Methods

### Recruitment and settings

Nurses (n = 26) from 11 inpatient units at an academic tertiary and two community hospitals were recruited to participate in the NEAT study, which took place over a 9-month period. Nurse leadership at the respective institutions were presented with the program and asked to identify hospital units where nurses were routinely expected to educate patients with DM. This project was approved as a Quality Improvement project (Project #0001512) by the UPMC Quality Review Committee.

### Phase 1: Exploratory focus groups

A series of focus group meetings were scheduled with registered nurses on various units to explore nurse insights regarding educating hospitalized patients with DM. Trained members of the research team presented scripted questions and examples of current education materials, including hand-outs, videos, and knowledge-based questionnaires to the nurses. Nurses were asked to provide their opinion about inpatient diabetes education in terms of barriers, content, delivery and support mechanisms. Nurse responses were transcribed and analyzed by trained members of the research team to identify emerging key themes.

### Phase 2: Designing and evaluating NEAT

Focus group findings identified key elements (Table 1) and informed development of a structured delivery protocol (Table 2) and tools to guide staff nurses in providing patient-centered “survival skill” education to assure safety after hospitalization and transition to existing outpatient diabetes education. Easy to use patient assessments were developed to capture information that is in accordance with the National Standards for DSME/S and DSME program recognition [23, 24]. In collaboration with nurses, the research team developed and/or selected already available brief, short video vignettes on key diabetes topics: nutrition, activity, insulin administration, injection techniques, and risk reduction in regards to hypo- and hyperglycemia. Video content aligned with guidance from the American Association of Diabetes Educators’ “AADE7 Self-Care Behaviors™” [25] and was based on the feedback from the nurse focus group discussions. Videos were uploaded onto iPads in order to provide a user-friendly, efficient mechanism for education delivery. In addition, “teach back” tools, a standardized approach to diabetes education for electronic medical record (EMR) documentation across internal institutions and units, and a discharge survival skills summary sheet were designed. Nurses were identified on specific hospital units to deliver NEAT and trained to schedule outpatient DSME visits for patients prior to hospital discharge. Nurses were instructed to follow the protocol to implement NEAT in their respective units with those patients who they identified in need of education and able to participate. During the course of program implementation, the research team provided study and technological support to the participating nurses. After a period of use, during which the NEAT model was used to deliver education to approximately 25 patients, the research team held focus groups with staff at each of the participating hospitals to assess their experiences using NEAT, with particular attention to its usefulness in meeting the needs of staff related to the delivery of diabetes survival skill education.

**Table 1:** NEAT key elements

• Brief video vignettes focused on diabetes self-management “survival skills”
o Nutrition o Medication taking o Insulin injections o Blood glucose monitoring o Hypoglycemia
• Patient knowledge assessment • Nurse “cheat” sheet to aid in patient knowledge acquisition • Survival skills take home sheet • Diabetes education resource list to aid in scheduling outpatient visits prior to discharge • Uniform documentation guidance in electronic medical record

**Table 2:** NEAT protocol

1. Assess patient diabetes self-management needs
2. Prioritize learning needs critical to assuring a safe transition to home, e.g., injection skills, identifying and treating hypoglycemia, emergency call numbers, etc.
3. Select appropriate videos accordingly.
4. Deliver and review video/iPad with patient
5. Assess knowledge through teach back with quiz
6. Provide patient/caregivers with “Survival Skill” take home sheet
7. Make appointment for diabetes educator on discharge
8. Document in the electronic medical record

## Results

### Phase 1

Staff nurses viewed teaching DM patients as part of their job, but reported barriers: lack of time/resources and guidance on expectations (“What are we expected to do and accomplish?”), shortened LOS and caring for sicker patients who require their attention for pain management and are often sedated. In addition the nurses’ shared their lack of confidence in providing accurate information on current therapies/tools and fear that patients will ask questions that they cannot answer, thus jeopardizing patient trust. Nurses agreed that education was important and should be designed to assure safety after discharge, focusing on “survival skills” related to hypoglycemia, medication, nutrition, and blood glucose monitoring, and directing patients to outpatient DSME. They advised that DSME should be patient-centered, targeted, assessment-based and user-friendly to accommodate sicker patients and health literacy. Nurses recommended developing brief videos with iPads or similar technology to facilitate delivery of survival skill education. Ideas for supporting staff nurses were access to a dietitian; resource nurse and/or centralized diabetes educator (most reasonably the hospital outpatient educator) for more complex cases; easily accessible, routinely updated, to-the-point web-based information and incentives for maintaining diabetes-related competencies. Nurses encouraged EMR enhancements to simplify charting, provide survival skills discharge educational tools and improve care coordination with outpatient DSME services.

### Phase 2

Nurses who participated in the delivery of NEAT reported that the program met the goal of providing patient-centered diabetes survival skills, particularly for introducing patients to therapies and devices and reviewing dietary considerations. They reported that the videos embedded on the iPads provided a convenient and standardized method for facilitating teaching at the bedside. All agreed that a video modality was a useful mechanism for meeting the needs of hospitalized patients who are unwell and those with health literacy issues. The video format also afforded the opportunity for patients to view and review educational presentations at times convenient for them. No problems were reported with patient’s ability to use the iPad technology and several nurses found that patients reported liking this modality. Moreover, the NEAT approach relieved pressure from the staff nurses’ workload. Overall, nurses found the protocol easy to follow and commented that it allowed for a standardized, yet tailored approach to survival skill education. Some challenges and opportunities for improvement were noted too. Nurses thought it would be beneficial to add an interactive feedback mechanism to the video to allow patients to self-assess knowledge after viewing videos. They also thought it would be beneficial to engage caregivers when appropriate. In addition, most found it challenging to schedule visits with an outpatient educator prior to discharging patients, with the most frequently cited issue being uncertainty as to whether the education would be covered by the patient’s insurance plan.

## Discussion

It is often the case that health care services are recommended and expected to be provided without gaining insights from the very people who are presumed to deliver them. In this report, we demonstrate that NEAT – developed based on information gathered from nurses who are expected to deliver inpatient DM education - is a useful program for bedside nurses in providing survival skill education for the hospitalized patient with DM. Bedside nurses found that the NEAT process and tools met the majority of the basic diabetes education needs of their patients while relieving their workload.

This study also reaffirms challenges that bedside nurses’ face [15, 16] and identifies opportunities and strategies for improving content and delivery. Although bedside nurses do see patient education as part of their job, competing demands, high acuity level of patients now admitted to the hospital, their limited self-knowledge regarding new therapies and tools and confusion regarding teaching expectations serve as barriers to successfully carrying out this responsibility. Increasingly, hospital LOS is becoming shorter with discharge dates often determined by payers. This creates additional problems in planning for the delivery of inpatient education when predetermined discharge schedules are not made available. Nurses facing these challenges do appreciate diabetes educators and dietitians as resources, believe that technological approaches should be explored and that comprehensive DSME is essential but should occur in the outpatient setting. The nurses’ message is consistent with others who find that providing diabetes education for safe transition to home is critically important and [15, 18, 19, 26, 27] given the large number of hospitalized patients with diabetes, the focus for inpatient education will need to be on survival skill training [15, 18].

Findings from this study also corroborate those of a previous qualitative study where investigators exploring causes for readmission in people with DM found reoccurring themes that emerged as contributors to readmission risk [28]. Themes that can be addressed through DSME included patient poor health literacy (lack of knowledge about diabetes and discharge instructions); failure to follow discharge instructions; lack of awareness of medication changes, limited social support; and loss of control over illness. To reduce the readmission risk for DM patients, the investigators recommended that survival skills education address sick day care and recognition, treatment, and prevention of hyperglycemia and hypoglycemia, as well as the logistics of taking diabetes medications [28]. They reiterated the need for staff nurses with discharge instruction to refer to outpatient DSME that offers the advantages of ongoing management, inclusion of family support, a teachable moment and a patient-centered approach.

Given that the number of people who receive DSME and provider referrals continues to be low, innovative approaches to promote follow up DSME services are necessary [29, 30]. A recent position statement jointly issued by the American Diabetes Association, the American Association of Diabetes Educators, and the Academy of Nutrition and Dietetics [10] acknowledged the need for a systematic referral process to promote uptake of DSME services. To this end, an evidence-based diabetes education algorithm was developed to provide guidance on when, what, and how DSME/S should be provided. As might be expected, DSME is recommended when factors present that may influence self-management, including a transition from hospital to home. Mechanisms to assure automatic referrals to DSME, possibly through EMRs, upon hospital discharge need to be improved. In addition, physicians working in hospitals like hospitalists need to be informed about referrals and the benefits of outpatient DSME.

In addition, resources need to be directed to support the use of technology. In another study of inpatient education, educating hospitalized patients about warfarin by using a video on an iPad was shown to be effective [31]. The pharmacists leading this study conclude that video education on an iPad may be an alternative to traditional education in the hospital setting. Nurses in the NEAT study also support the use of iPads as a technology-based approach for delivery.

Like other hospital health systems facing the challenge of a growing DM population and inverse number of available certified diabetes educators (CDEs) and resources [18, 32], the study institution has implemented a system-wide model whereby trained diabetes resource nurses focus on providing staff education and assessing competency, while staff nurses educate the patient on basic skills and content during the hospital stay. The ultimate goal is to use “precious” education resources wisely. CDEs, who also serve the outpatient population, are charged with providing support and training for hospital staff on new diabetes therapies, protocols, education strategies and for those inpatients identified to have more challenging/complex diabetes education needs. At the time of discharge, the aim is that patients have received education on survival skills and are connected to follow-up in outpatient medical management and outpatient education. Tracking actual participation in the DSME service is underway.

The limitations of this project are recognized. NEAT is a qualitative feasibility study designed to elicit reactions from nurses. Findings are representative of nurses who work within the same hospital system, although the nurses did represent academic and community- based hospitals. The study did not include a control group, as it would have been unethical to deny diabetes education to patients [33]. Study obligations and timelines limited opportunity for large scale patient recruitment and assessment of patient-level outcomes. The NEAT implementation was dependent on the availability of current nurse staffing and patient loads. For example, engaging nurses when they were caring for a full patient load was difficult and limiting. Recognition is also given to the need to attend to psychosocial issues. For example, distress levels are reported to be high at DM diagnosis [34]. This should apply to those newly diagnosed during a hospitalization. Acuity and efforts to address patient needs such as pain management are often the priority of bedside nurses during hospitalization. However, there may be opportunities to integrate methods for assessing and addressing psychosocial issues, at least in a limited capacity, and this should be explored in future research.

## Conclusions

With the growing number of people with DM and their need for self-management education, health systems would be wise to consider programs that address the needs of hospitalized patients with DM and the staff that are expected to provide their care. Messages from front line nurses providing these services need to be taken seriously. Hospital leadership should set clear expectations that inpatient education for patients with DM be funneled to the provision of survival skill education. Nurses with competing demands and limited opportunities for training on diabetes–specific topics should not be expected to provide comprehensive DSME. Those keen to prevent hospital readmissions, should strongly consider improving resources and access to outpatient diabetes education. The need for evidence-based standardized inpatient education processes is warranted.
